# Medicines for children: flexible solid oral formulations

**DOI:** 10.2471/BLT.16.171967

**Published:** 2017-03-01

**Authors:** Ebiowei SF Orubu, Catherine Tuleu

**Affiliations:** a School of Pharmacy, University College London, 29–39 Brunswick Square, WC1N 1AX, London, England.

Children younger than five years old are generally unable to safely swallow solid capsules and tablets larger than 10 mm.^1^ Although oral liquid medicines can be prescribed, these have some disadvantages over solid medicines. Substances or excipients that solubilize the active ingredient or ensure microbial stability are included in oral liquids and these may be harmful to young children.^2^ Liquid medicines tend to be more expensive than solid medicines and this makes them less accessible to patients who pay for medicines out-of-pocket. Another problem is that liquid medicines are less chemically stable than solid medicines and require refrigeration in hot climates to guarantee their quality and efficacy. In countries where liquid formulations are not available, caregivers may manipulate solid medicines to make them easier to swallow, thus jeopardizing the quality, safety and efficacy of the medicine. These issues are a concern in global efforts to improve access to age-appropriate essential medicines for young children, especially in low- and middle-income countries.^3^

The challenges of ensuring access to suitable medicines for children led the World Health Organization (WHO) in 2008 to propose flexible solid oral dosage forms as the preferred formulations for children.^4^ These are solid forms that do not have to be swallowed whole, such as dispersible tablets, effervescent tablets, chewable tablets, orodispersible tablets and sprinkle capsules. These formulations potentially solve the problems of safety, cost and storage of liquid medicines. Better access to age-appropriate medicines is especially important for low- and middle-income countries, which have the highest burden of under-five mortality from preventable and treatable diseases.^5^ However, the barriers to implementation of flexible solid oral dosage forms in these countries are not well understood. Three factors need to be considered: (i) country needs, (ii) local manufacturability, and (iii) acceptability to users.

First, we need to know which oral medicines are currently available in easy-to-swallow forms. Knowledge of which new formulations are needed in low- and middle-income countries would help drive medicines’ development.

**Fig. 1:**
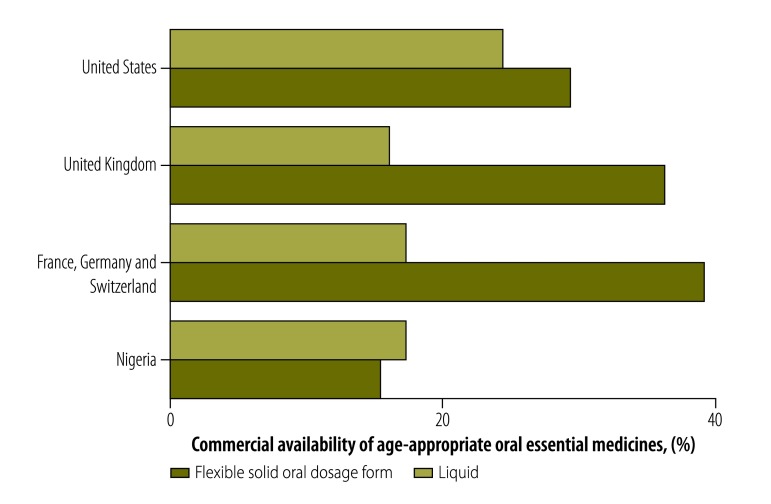
Commercial availability of liquid and flexible solid oral dosage forms of 143 essential oral medicines in six countries, October 2014

Notes: Availability was expressed as a percentage of the oral medicines (*n* = 143) on the *WHO model list of essential medicines for children* (3rd list, March 2011).^7^ France, Germany and Switzerland were grouped because their geographical placement suggests that medicines available in one country could be readily accessible to the other two countries. Flexible solid oral dosages included dispersible tablets, effervescent tablets, chewable tablets, orodispersible tablets and sprinkle capsules, all of which are solids that can be administered dispersed in liquids or on soft foods. Crushable tablets were also included, even though technically, these may not be considered flexible solid oral dosages.

In 2014, a needs assessment was made in selected countries to determine which oral medicines in the WHO essential medicines list for children (3rd list, 2011) were available in age-appropriate forms.^6^ Nigeria, as an exemplar lower-middle-income country, was compared with five high-income countries who are leaders in the production of innovative medicines. The commercial availability of flexible solid oral dosage forms and liquid forms was determined from each country’s compendium of pharmaceutical products (Fig. 1). Of 143 oral essential medicines, only 22 (15%) were available as flexible solid oral dosage forms in Nigeria compared with 56 (39%) in France, Germany and Switzerland combined.

In the short term, regulatory efforts (for example, reduced cost of, and faster, product registration) may be needed to facilitate the import of the available flexible solid oral dosage forms into countries lacking these child-friendly forms. The recent push by WHO for regulatory harmonization among low-income countries in Africa is a step in the right direction.^8^ The inclusion of age-appropriate dosage forms in national formularies would also be necessary.

Second, to ensure sustainability of access in the long term, countries need to be able to manufacture flexible solid oral dosage forms in a cost-effective manner. Several low- and middle-income countries in Africa are getting ready to manufacture medicines under the WHO pre-qualification programme which aims to provide quality assurance. Some countries (e.g. Morocco, Nigeria, South Africa and the United Republic of Tanzania) currently manufacture quality-assured products including dispersible zinc tablets. This implies many countries have the local capacity to manufacture child-friendly medicines.

To further realize this potential, product development of flexible solid oral dosage forms needs to become cheaper in lower-income countries. One way would be to develop a technology platform for the production of dispersible tablets in which active pharmaceutical ingredients of oral essential medicines can be fitted interchangeably. To facilitate this, pharmaceutical companies could collaborate with local academic institutions to develop or optimize formulations. The planning needs to take account of the material and physicochemical properties of the active pharmaceutical ingredients to determine their suitability for production by the comparatively low-cost direct compression tabletting technique. Oral active pharmaceutical ingredients have varying properties and some pose challenges to manufacturing by this method under standard conditions. Predictive tools to identify and solve problems in advance of manufacturing are needed.

Such tools are being developed. In 2015, the Manufacturing Classification Systems Group, in association with other working groups of the Academy of Pharmaceutical Sciences, published a proposal for a drug product manufacturing classification system. This aims to match a manufacturing method with an active pharmaceutical ingredient according to the ingredient’s material properties.^9^ This predictive tool can simplify and guide decision-making in the manufacturing of flexible solid oral dosage forms. A second tool is a table of excipients that could guide the selection of excipients to compensate for undesired material properties of an active pharmaceutical ingredient, such that the active ingredient is rendered suitable for manufacturing by direct compression methods.^10^ Compiling these tools into a single expert system that would also incorporate the physicochemical properties of the active ingredients of essential medicines could reduce the cost of manufacturing dispersible tablets. Medicines that cannot be produced as dispersible tablets can be formulated into other flexible solid oral dosage forms. These include chewable tablets, which can be given to children from the age of two years, and multi-particulates, which are administered directly into the mouth or dispersed in water.

Greater cost savings could be made. Most low- and middle-income countries in Africa currently import the active pharmaceutical ingredients and equipment needed for the production of pharmaceuticals. As such, currency exchange fluctuations, and problems in sourcing foreign currency to pay for imports, can be a hindrance to local production of pharmaceuticals. For such countries, national governments could grant tax remission for pharmaceutical raw materials and machinery. Ultimately, though, these countries would need to develop the capacity for the local production of active pharmaceutical ingredients and machinery. This way, higher cost savings can be realized. If the cost savings were passed on to the patient, access to medicines could be improved for patients who are not covered by reimbursement or insurance schemes, as in most low- and middle-income countries.

A third factor to consider is whether a change from liquids to dispersible tablets is acceptable to end-users (caregivers mostly, but also prescribers and dispensers). Acceptability of a dosage form and the ability of end-users to administer it as intended improves users’ adherence to treatment and hence the efficacy of the drug. A study in Kenya comparing two antimalarial drugs found that the dispersible form was simple for patients to use.^11^ Nevertheless, possible barriers to use of flexible solid oral dosages are still not much studied. Literacy levels, poor understanding of dosage forms, belief systems about medicines, personal preferences and access to water can all affect users’ acceptability of a dosage form, as does the cost of different formulations.

The palatability (taste, after-taste, grittiness, smell) of flexible solid oral dosage forms needs to be considered early on in product development. Palatability is an important factor in the acceptability of medicines in children, although this can usually be achieved with the use of sweetening and flavouring agents. When a company manufactures a product for different regions it may be necessary to adapt flavours to different regional preferences.

Caregivers’ perceptions would also need to be addressed. For children younger than five years, the available literature suggests that caregivers prefer liquid formulations despite the disadvantages of cost and stability mentioned earlier.^12^ Where the end-user is unwilling (or unable) to use dispersible tablets because of a preference for liquid medicines, the advantages of facilitating access to flexible solid oral dosage forms would not be achieved. Educational strategies for caregivers would need to be designed to emphasize the benefits of a change from liquids to flexible solid forms for this age group.

Access to safe water remains a concern in many low-resource countries. The use of milk or food to administer dispersible tablets ‒ to improve palatability or when safe water is unavailable ‒ is little studied. The compatibility of administered medicines with these different media is also not known. One option is for manufacturers to consider including plastic vials of drinking water with dispersible medicines in certain countries.

Clinical studies are needed to guide dosing and administration of flexible solid oral dosage forms in children. The ethical barriers to clinical trials in children, and the poor financial returns from investing in this relatively small proportion of patients, are being addressed by recent regulations making such trials mandatory in children and providing financial incentives for doing so.^13^ However, the challenge of obtaining statistically valid data from small paediatric populations still remains. Computer modelling of drug pharmacodynamics and pharmacokinetics to generate data for safety and efficacy studies in clinical medicines’ development is being considered by regulatory agencies.^13^^,^^14^

In conclusion, national governments and manufacturers need to work with WHO to resolve the potential challenges of promoting the use of flexible solid oral dosage forms for children.
